# Study on oscillation effect of wind power pile foundation on local scour

**DOI:** 10.1038/s41598-025-02934-8

**Published:** 2025-05-29

**Authors:** Zhenzhou Zhao, Chunhao Su, Yan Liu, Yige Liu, Huiwen Liu, Yuanzhuo Ma, Shangshang Wei, Shijun Li

**Affiliations:** 1 Key Laboratory of Wind and Solar Energy Utilization Technology, Ministry of Education, Inner Mongolia University of Technology, Hohhot, 010051 China; 2 College of Renewable Energy, Hohai University, Changzhou, 213200 China

**Keywords:** Offshore wind turbine, Scour, Pile oscillation, Horseshoe vortex, Bed shear stress, Renewable energy, Wind energy

## Abstract

The pile-oscillation effect on foundation local scour cannot be neglected in the large-scale offshore wind turbine. To reveal the fluid dynamics and its influence on the bed shear stress of an oscillating pile foundation, the VOF two-phase flow model is used to study the effect of the transverse and vertical oscillation of pile foundations employed in offshore wind turbines. The changes in the flow field, horseshoe vortices, and bed shear stress are analyzed under different oscillation frequencies and amplitudes are monitored by two symmetry planes ahead of the pile and at pile side respectively. The results show that, at the longitudinal oscillation condition, the backflow in front of the pile is produced and is strengthened as increasing the frequency and amplitude of oscillation; the size of horseshoe vortex ahead of the pile, and the bed shear stress reach the maximum at the moment of *T*/2 (*T* is the oscillation cycle). At the transverse oscillation, horseshoe vortices are extended to the pile side, their sizes are increased as the oscillation frequency and amplitude increase, and the maximum size is generated at the moment of *T*/2; while the bed shear stress is the smallest at *T*/2, and the maximum is created at *T*. Both transverse and longitudinal oscillations increase the time-averaged bed shear stresses (TBSS), the longitudinal oscillation generates larger TBSS in the symmetry plane in front of the pile, conversely transverse oscillations generate larger TBSS in the side symmetry plane of the pile.

## Introduction

The monopile foundation is widely used for offshore wind turbines due to its simple structure, economical space occupation, and wide applicability^1–4^. The pile foundation causes a dramatic change in the structure of seawater flow around it, which results in increasing the shear near the bed and forming localized scour pits that threaten the safety of the unit^5,6^. The scour depth is one of the most important engineering parameters in offshore wind farm design^7,8^.

The vortices around the pile foundation are the direct cause of scour. Numerous studies have explored the mechanism of local scouring of bridges^9–14^. Most of these studies deduced that in the boundary layer, the water flow is impeded by the pile foundation, which results in reducing the flow rate, and thus the downstream pressure is large and the upstream pressure is a small counterpressure gradient. In addition, when the counterpressure gradient is large to a certain extent, the original movement of the fluid is forced to stop and move downstream under its action. This results in the formation of a horseshoe vortex system in the corner area of the ground close to the pile foundation, which is the main reason for the formation of local scouring. Roulund et al.^15^ conducted experiments and numerical simulations on a monopile foundation under constant water flow conditions. They deduced that the flow lines on the two sides of the pile foundation are squeezed and constricted, which results in increasing the flow velocity as well as increasing the bed shear stress on the pile side by a factor of approximately ten. Margheritini et al.^16^ studied the local scouring of pile foundations under tidal currents. They deduced that the shape of the scour pits upstream and downstream a monopile foundation is roughly symmetrical due to periodic variations in the flow velocity. Sumer et al.^17,18^ studied localized scour under wave conditions and obtained equations for predicting its depth in a live bed. Sumer and Fredsøe^19,20^ studied the local scouring of monopiles under combined current and wave conditions. They deduced that the vortex field consists of three main components: horseshoe vortex in front of the pile foundation which has streamline constriction on the two sides of the monopile, vortex shedding flow downstream of the monopile, and local scouring around the pile foundation caused by the vortex flow. It is important to mention that all the aforementioned studies tackle stationary pile foundations as they aim at analyzing the horseshoe vortex system and scour mechanism by changing the water flow conditions.

The pile foundations are subjected to cyclic loading by wind, waves, currents, and blade rotation, generating vibrations in the longitudinal and transverse directions, which affects the local scour. Therefore, the local scour of the pile base is determined by the coupling of the pile base oscillation intensity and hydrodynamic conditions, at which time two different scour modes exist around the oscillating pile base. The first mode exhibits an inverse relationship between the equilibrium scour depth and the oscillation intensity. Li et al.^21^ conducted a three-dimensional experimental study on the time evolution of the convective region around a vibrating pile foundation. Their results showed that increasing the force or the loading frequency allows to increase the time development and the final size of the settling bed. Guan et al.^22^ and Al-Hammadi and Simons et al.^23^ studied the local scour on monopile foundations under cyclic longitudinal loading and water flow conditions based on flume experiments. They deduced that the initial development of local scour under cyclic longitudinal loading is faster than in the scenario which does not include cyclic lateral loading, but the scour depth is reduced. When the cyclic lateral load is unloaded, the scour depth exceeds that of the scenario which does not include cyclic lateral loading, and it is affected by the structural vibration^23,24^. .Al-Hammadi and Simons^23^ attributed the shallower scour pits around the vibrating monopile foundations to the cyclic lateral vibration resulting in the continuous backfilling of sand to the bottom of the scour pits. Guan et al.^22^ attributed the same phenomenon to the densification and convective movement of sand around vibrating monopile foundations. Another scour pattern presents a positive correlation between the equilibrium scour depth and the vibration intensity. Yao et al.^25^ showed that the equilibrium scour depth of a static monopile foundation is less than that of a vibrating monopile foundation subjected to cyclic lateral vibration under weaker hydrodynamic conditions.

The mainstream methods for scour prediction include scalar equation method, discrete element method (DEM) and dynamic mesh bottom morphology tracking method, among these methods, the dynamic mesh bed morphology tracking method is more effective and suitable for engineering applications because it controls the changes of the riverbed boundary through bed deformation equations. However, this method lacks comprehensive consideration of the complex physical mechanisms of localized scour pits (Li et al.^26^), especially regarding the critical bed shear stress. When the bed shear stress exceeds the critical starting stress (defined by the Shields criterion) of the sediment, the sediment particles begin to detach from the bed, leading to the formation and expansion of scour pits.

The monopile foundations studied by previous literature are stationary, such as bridge foundations^9–20^. While, wind power pile foundations are always in lateral and longitudinal oscillations due to complex loads exerted by wind wave, and current. Although Guan, Al-Hammadi, Simons and Yao et al.^22–25^ mentioned above investigated the effect of the oscillation intensity of pile foundation on the local scour depth through experiments, the change of hydrodynamic conditions around the pile foundation due to the oscillation of the pile foundation has not been studied in depth, while the mechanism of the change of the bed shear stress around the pile foundation triggered by the change of hydrodynamic conditions is still not clear.

The monopile foundation of offshore wind turbines is researched considering the pile oscillation based on the OpenFOAM open-source software. The flow field, horseshoe vortices, and bed shear stress are analyzed from different oscillation frequencies and amplitudes. The distribution and changes of the three parameters in two symmetry planes in front of the pile and on the side of the pile are monitored. All in all, we deeply explore the local scour considering the pile oscillation to promote the understanding of scour mechanism of offshore wind turbine foundation working in the complex sea environment, and hope to fill the gap in this area.

## Numerical method

### Free liquid level simulation method

The VOF method is used to capture the gas–liquid two-phase flow cross-interface, which is governed by the following equations:

1 $$\frac{\partial \alpha }{{\partial t}} + \frac{{\partial \left( {u_{i} \alpha } \right)}}{{\partial x_{i} }} = 0$$

where *α* denotes the volume fraction (*α* = 0, *α* = 1, and 0 < *α* < 1 represent air, liquid, and gas–liquid interface, respectively).

In the equation solving process, the gas–liquid two-phase flow is treated as a mixed liquid whose physical properties can be expressed as:

2 $$\rho = \alpha \rho_{1} + \left( {1 - \alpha } \right)\rho_{2}$$

3 $$\mu = \alpha \mu_{1} + \left( {1 - \alpha } \right)\mu_{2}$$

where *ρ* denotes the density of the liquid mixture, *ρ*_1_ and *ρ*_2_ respectively denote the densities of the liquid and gas, *μ* denotes the kinetic viscosity coefficient of the liquid mixture, *μ*_1_ and *μ*_1_ denote the kinetic viscosity coefficients of the liquid and gas, respectively.

### Turbulence model

The SST *k*–*ω* turbulence model is a hybrid model combining the *k*–*ε* and *k*–*ω* models^27–29^. It can accurately capture the counterpressure gradient and separated flow^15^. Therefore, in this paper, the SST *k*–*ω* model is used to describe the counterpressure gradient generated in front of the pile foundation and the formed horseshoe vortex.

### Computational domain and meshing

**Fig. 1:**
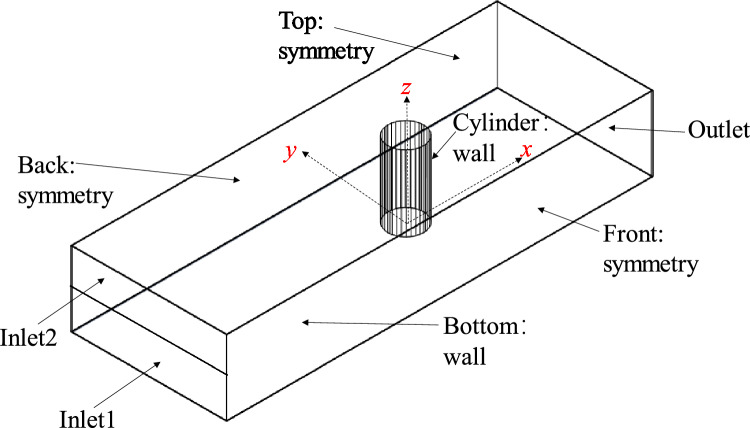
Boundary conditions.

The computational domain is shown in Fig. 1, where the center of the circle at the base of the pile foundation is placed on the coordinate origin, the transverse, longitudinal, and vertical directions follow *x*, *y*, and *z*, respectively. In order to facilitate the comparison with Roulund et al.^15^ cylindrical test data, the diameter of the pile base (*D*), the length of the flow field domain (*x*-direction), its width (*y*-direction), and height (*z*-direction) are set to 0.536, 35, 3, and 1 m, respectively. The depth of the water and the velocity of the incoming current (*U*_m_) are set to 0.54 m and 0.326 m/s, respectively. The calculation domain, which is the center of the pile base circle, is shown in Fig. 1.

The boundary conditions are shown in Fig. 1. The cylinder denotes the pile of offshore wind turbine. To simulate the offshore monopile foundation located on the seabed, and the water depth is set to *H* = 0.54 m. Two velocity inlets are set up, Inlet1 is the liquid-phase inlet, and Inlet2 is the gas-phase inlet. All the physical parameters were set to have a vertical pressure gradient of zero at the outlet, to ensure a stable outflow. The Bottom and Cylinder, as shown in Fig. 1 are set as no-slip wall boundaries. The vertical pressure gradient is set to be zero at the solid-wall boundary to ensure that the flux through the solid-wall boundary is zero. The vertical volume fraction is also set to be zero at the boundary. The boundary conditions of the turbulence kinetic energy, turbulence dissipation rate, and turbulence viscosity coefficient are modeled using the wall function. The Top, Back and Front faces, as shown in Fig. 1 are symmetry boundaries.

**Fig. 2:**
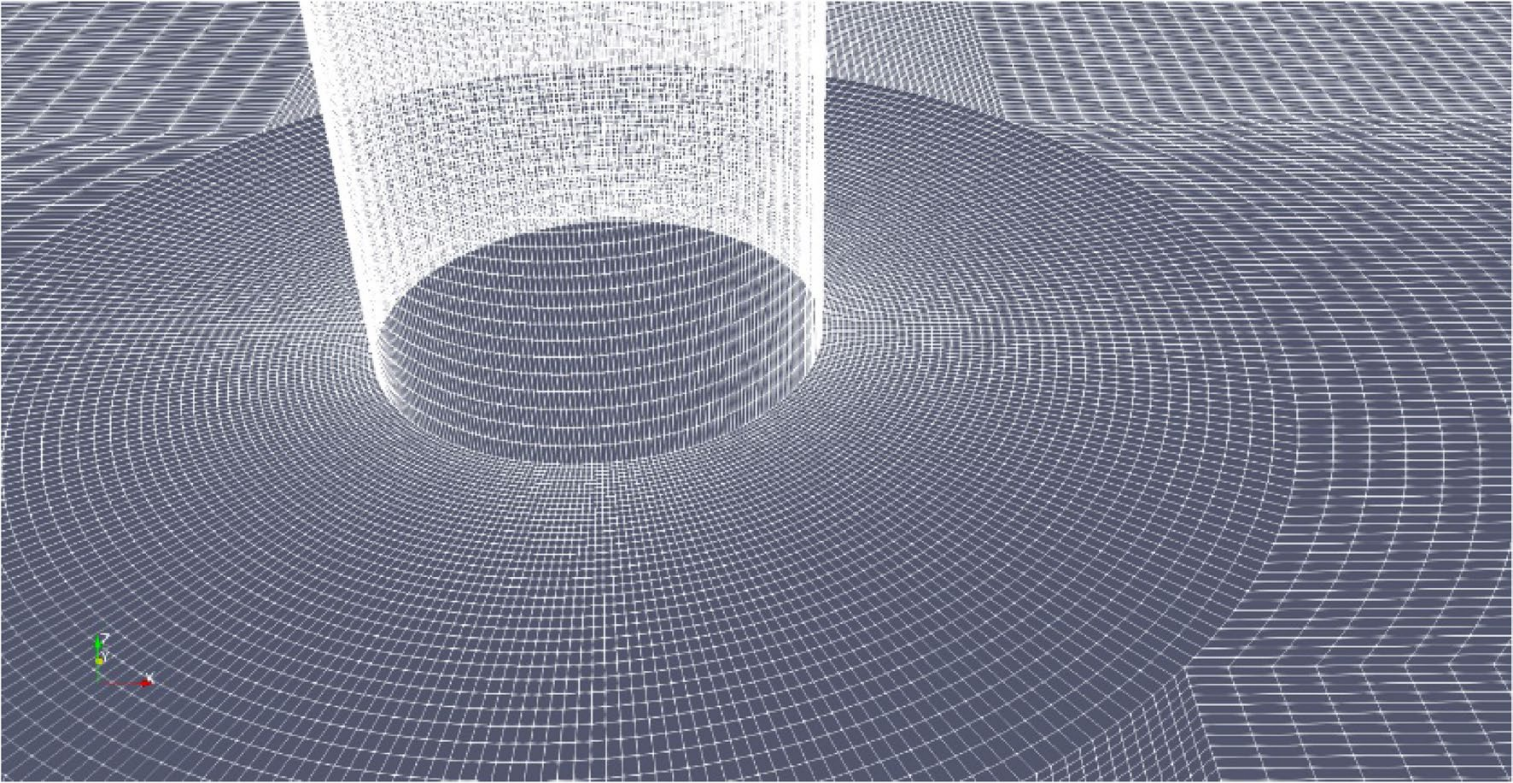
Local grid near pile foundation (this figure was generated using ParaView (version 5.11.1, URL: https://www.paraview.org)).

The hexahedral grids are used to mesh the zone around pile foundation, as shown in Fig. 2. The grid motion technique is used to model the moving solid boundary. OpenFOAM software provides two grid motion processing techniques, grid motion and grid topology change. We use the grid motion technique to cope with the cylinder (Fig. 1) oscillation to model the transverse longitudinal and vertical oscillations of the pile foundation. The finite volume method is used for spatial discretization and the time derivative term is discretized by integrating inside the control body. We use the Eulerian implicit time-difference method to discretize the convective term by integrating it inside the control body. In addition, we use the central difference format with second-order accuracy to interpolate the gradient and Laplace terms, use the van Leer format with second-order accuracy to interpolate the dispersion terms for the velocity and volume fractions, and use the windward format with first-order accuracy to interpolate the dispersion terms for the turbulence energy and energy dissipation rate.

### Model validation

**Fig. 3:**
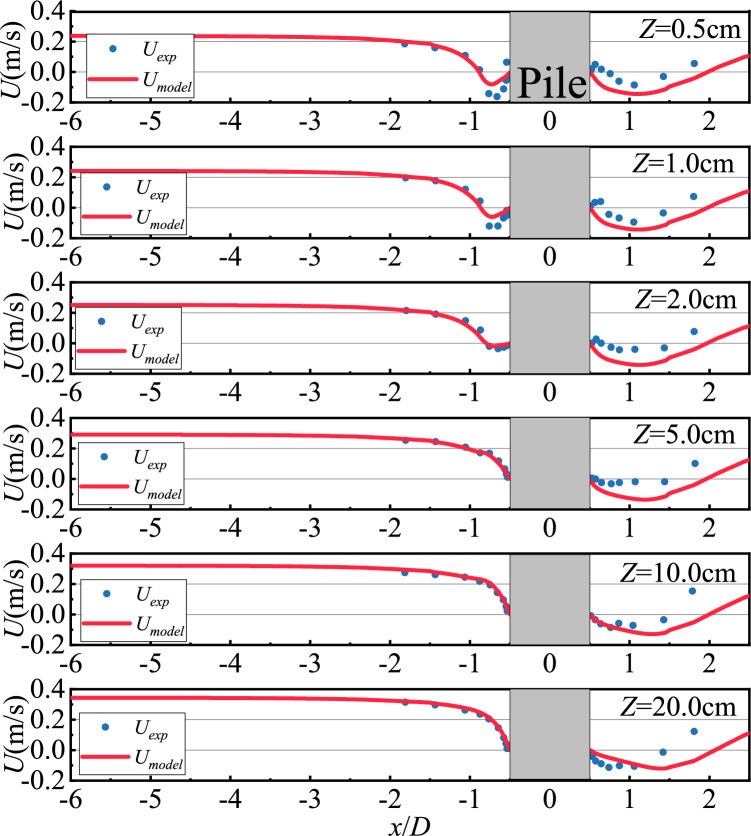
Horizontal flow velocity distribution diagram in the symmetry plane at different heights from the bed surface.

**Fig. 4:**
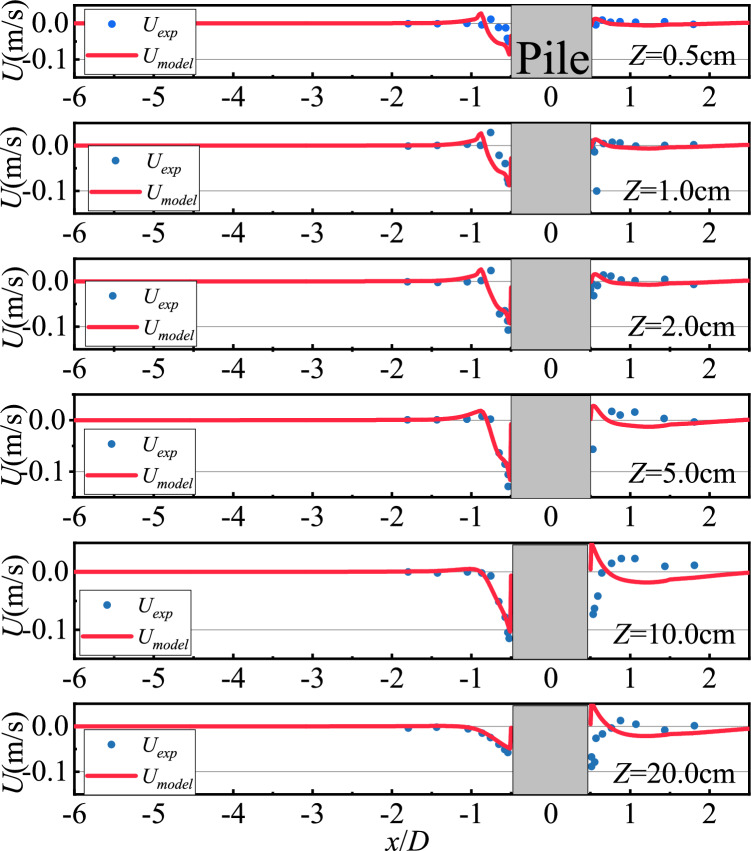
Vertical flow velocity distribution diagram in the symmetry plane at different heights from the bed surface.

The Roulund et al.^15^ cylindrical test data were validated against the stationary condition NS0 at 0.5, 1, 2, 5, 10, and 20 cm from the seabed surface and comparing them with the CFD values, as shown in Figs. 3 and 4. The numerical simulations in this paper also used flow velocities from a stationary pile bed for comparison with them. In the subsequent study, the main focus is on the data from an oscillating pile bed at different moments in a cycle. I consider this to be a process similar to discretization. The oscillating pile bed at different moments in a cycle is only a change in the position of the pile bed compared to the stationary pile bed, and there is no other essential difference, so I believe that the model validation for the non-oscillatory condition can be used for the oscillatory condition.

The modeled values of the horizontal flow velocity distribution at the upstream of the pile are mainly consistent with the test values, as shown in Fig. 3. At the downstream of the pile, the model flow velocity distribution is also consistent with the trend of the physical model test flow velocity, and the numerical difference is mainly caused by the variation of the vortex at the back of the pile base function of time. The modeled vertical flow velocity distribution in front of the pile is also consistent with the test values, as shown in Fig. 4. The overall flow velocity distribution behind the pile is consistent with the physical model test. In addition, the difference between the flow velocity distribution away from the bed and the physical model test is due to the fact that the smaller the water depth, the more intense the vortex movement at the back of the pile near its foundation, the vortex at the back of the pile base varies with time, and a reflux also exists. In summary, the CFD data fit well with the test values and the simulation method can be used for the flow field analysis below.

## Results

The amplitude and frequency settings presented in the study of Li et al.^21^ are adopted in this paper. The specific working conditions used in the calculation are shown in Table 1.

**Table 1:** Calculation conditions.

Case name	Direction of oscillation	*A* amplitude (mm)	*F* frequency (Hz)
NS0	None	0	0
XA5f2	Vertically	*A* = 5	*f* = 2
XA5f4		*A* = 5	*f* = 4
XA10f2		*A* = 10	*f* = 2
YA5f2	Transverse	*A* = 5	*f* = 2
YA5f4		*A* = 5	*f* = 4
YA10f2		*A* = 10	*f* = 2

**Fig. 5:**
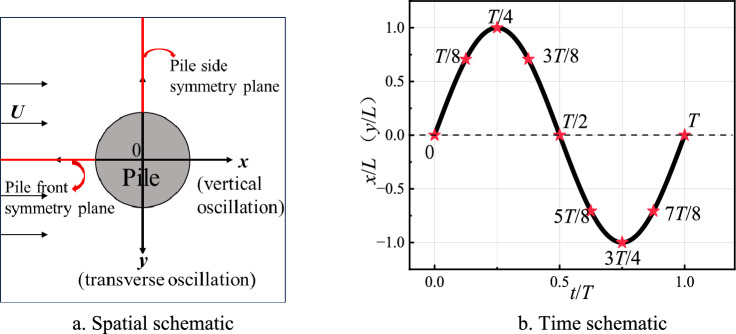
Schematic diagram of oscillation motion.

The pile oscillations generate cyclic loading, and the flow field and bed shear stresses show periodic transformations with the oscillations of multiple cycles. Therefore, in this paper, one oscillation cycle is selected to analyze the flow field and shear stress around the pile foundation. This study mainly focuses on the vortex field in the two symmetry planes of the pile front and side, as well as the magnitude of their bed shear stress, as shown in Fig. 5.Nine moment points during the oscillation period are selected to analyze the vortex field, as shown in Fig. 5b.

### Vortex structural analysis of stationary pile foundations

**Fig. 6:**
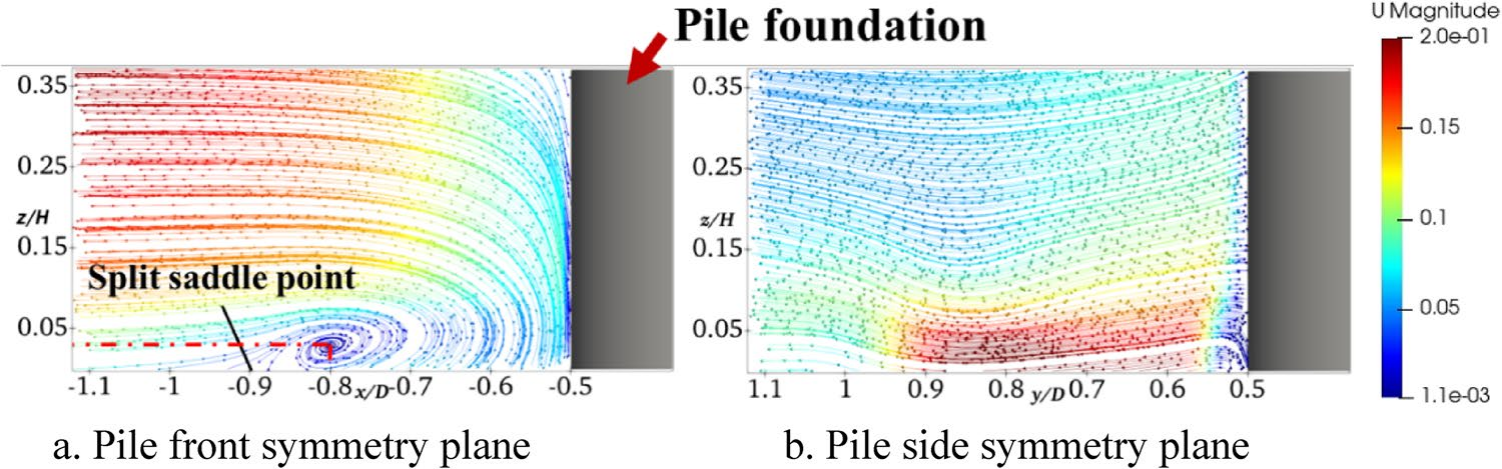
Streamline diagrams of NS0 (This figure was generated using ParaView (version 5.11.1, URL: https://www.paraview.org)).

Figure 6 shows the streamlines in the symmetry plane in front of the pile and on its side at rest. The horseshoe vortex can be clearly seen in Fig. 6a, where the center of the vortex nucleus is located at *x*/*D* = −0.8 in front of the pile and *z*/*H* = 0.025 on the upper side of the bed. As the pile base impedes the incoming flow and forms a descending flow near it, the horseshoe vortex is formed. A clear separation saddle point occurs near the bed surface around *x*/*D* = −0.9 in front of the pile. The activity range of the horseshoe vortex is between the separation saddle point and the pile base, and the main range of local scour is in front of the pile. It can be seen from Fig. 6b that there is no obvious horseshoe vortex within the symmetry plane on the pile side. However, the horseshoe vortex system gradually develops around the pile base, and the flow velocity is significantly larger than the upper water flow within *z*/*H* = 0.1 due to its impact, forming an accelerated flow. This is the main reason for the localized scouring on the pile side.

### Vortex structural analysis of longitudinally oscillating pile foundations

**Fig. 7:**
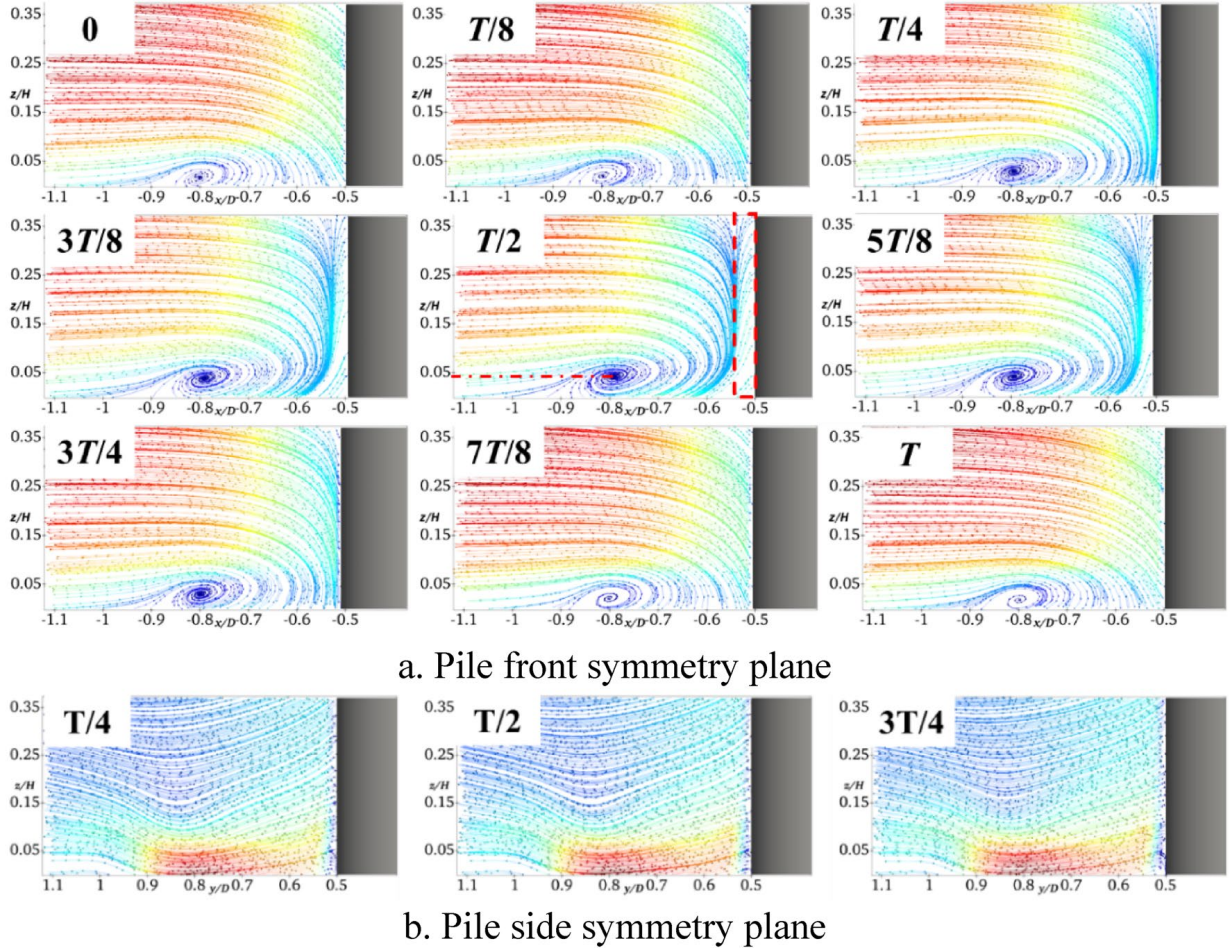
Streamline diagram of XA5f2 at different times (this figure was generated using ParaView (version 5.11.1, URL: https://www.paraview.org)).

It can be seen from Figs. 6a and 7a that, when the pile foundation undergoes longitudinal oscillation with *A* = 5 mm and *f* = 2 Hz, the vortex field in the symmetry plane in front of the pile is significantly changed. At *T*/4, there is a large water surface difference in front of the pile, which results in increasing the falling water flow, while the location and size of the horseshoe vortex basically remain unchanged. When the pile foundation oscillates at the pile front to the moment of *T*/2, there is a reverse water flow in front of the pile at *x*/*D* between − 0.5 and − 0.55. This forms a large amount of falling water flow by converging with the free-flowing water. In addition, the horseshoe vortex is sufficiently supplemented, the vortex nucleus center is raised to *z*/*H* = 0.04, its topology reaches its maximum value of 3*T*/4, and thus the topography is changed. Moreover, there is no obvious return flow in front of the pile and the horseshoe vortex decreases to *z*/*H* = 0.025. By comparing Figs. 6b and 7b, it can be deduced that the longitudinal oscillations do not intensify the degree of extrusion of the pile foundation on the pile-side water flow, and there is a slight change in the water flow in the symmetry plane on the pile side.

**Fig. 8:**
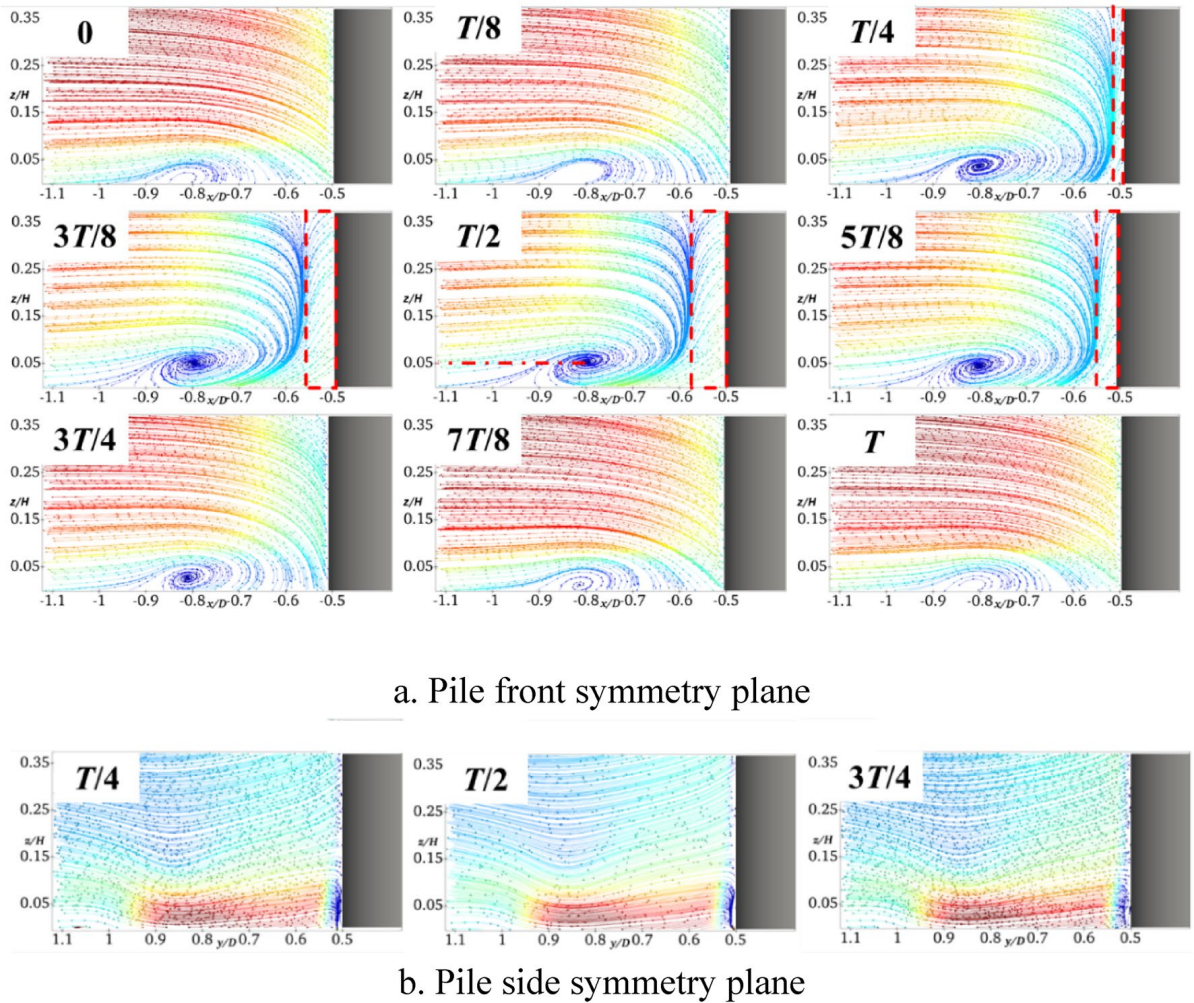
Streamline diagram of XA5f4 at different times (this figure was generated using ParaView (version 5.11.1, URL: https://www.paraview.org)).

Figure 8 shows the results obtained by setting *A* to 5 mm and increasing *f* to 4 Hz. It can be observed from Fig. 8a that there is an obvious reflux within *x*/*D* = −0.51 in front of the pile at the moment of *T*/4. This is mainly due to the viscosity of water. That is, when the oscillation frequency increases, the free incoming flow fails to replenish the water surface difference in front of the pile in a timely manner. In addition, the water flow on the pile side replenishes the water surface in front of the pile in a forward direction to form a reflux. It can be clearly seen that the reflux area is enlarged to *x*/*D* between − 0.5 and − 0.58 at the moment of *T*/2, the center of the horseshoe vortex rises to *z*/*H* = 0.05, and the flow velocity on the near bed surface flow velocity increases. By comparing Figs. 7b and 8b, it can be deduced that, when the frequency of the longitudinal oscillation increases, the symmetric surface water flow on the pile side is basically not affected.

**Fig. 9:**
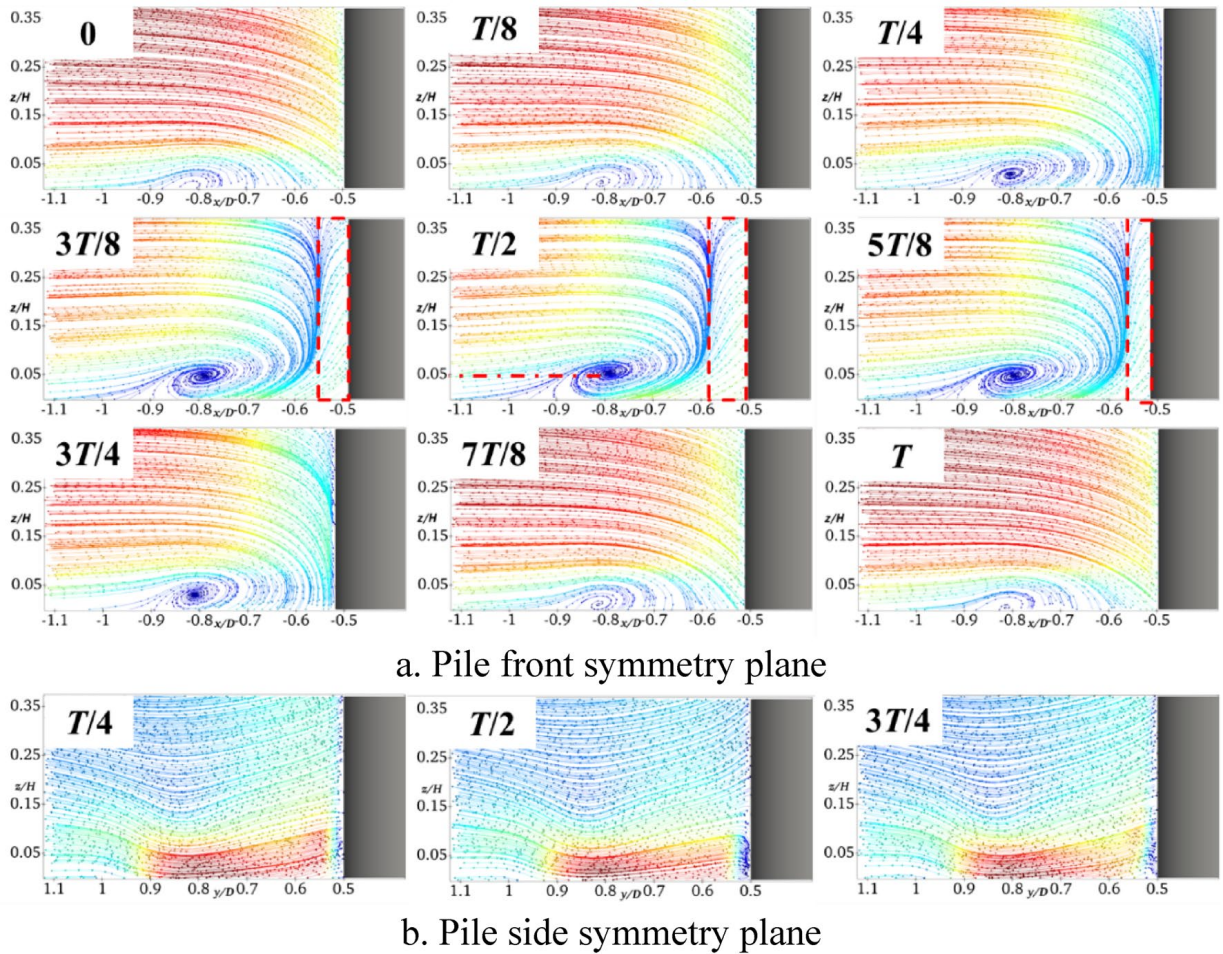
Streamline diagram of XA10f2 at different times (this figure was generated using ParaView (version 5.11.1, URL: https://www.paraview.org)).

Figure 9 shows the results obtained when setting *f* to 2 Hz and increasing *A* to 10 mm. By comparing Fig. 7a and Fig. 9a, it can be deduced that similar to the case of increasing the frequency, the reflux region at the moment of *T*/2 is increased to *x*/*D* between − 0.5 and − 0.58, the horseshoe vortex topology is increased, its center is raised to *z*/*H* = 0.05, and the flow velocity near the bed surface is also increased. By comparing Figs. 7b and 9b, it can be deduced that by increasing the amplitude of the longitudinal oscillation, the symmetric surface of the pile side is basically unaffected by the water flow.

### Vortex structural analysis of transversely oscillating pile foundations

**Fig. 10:**
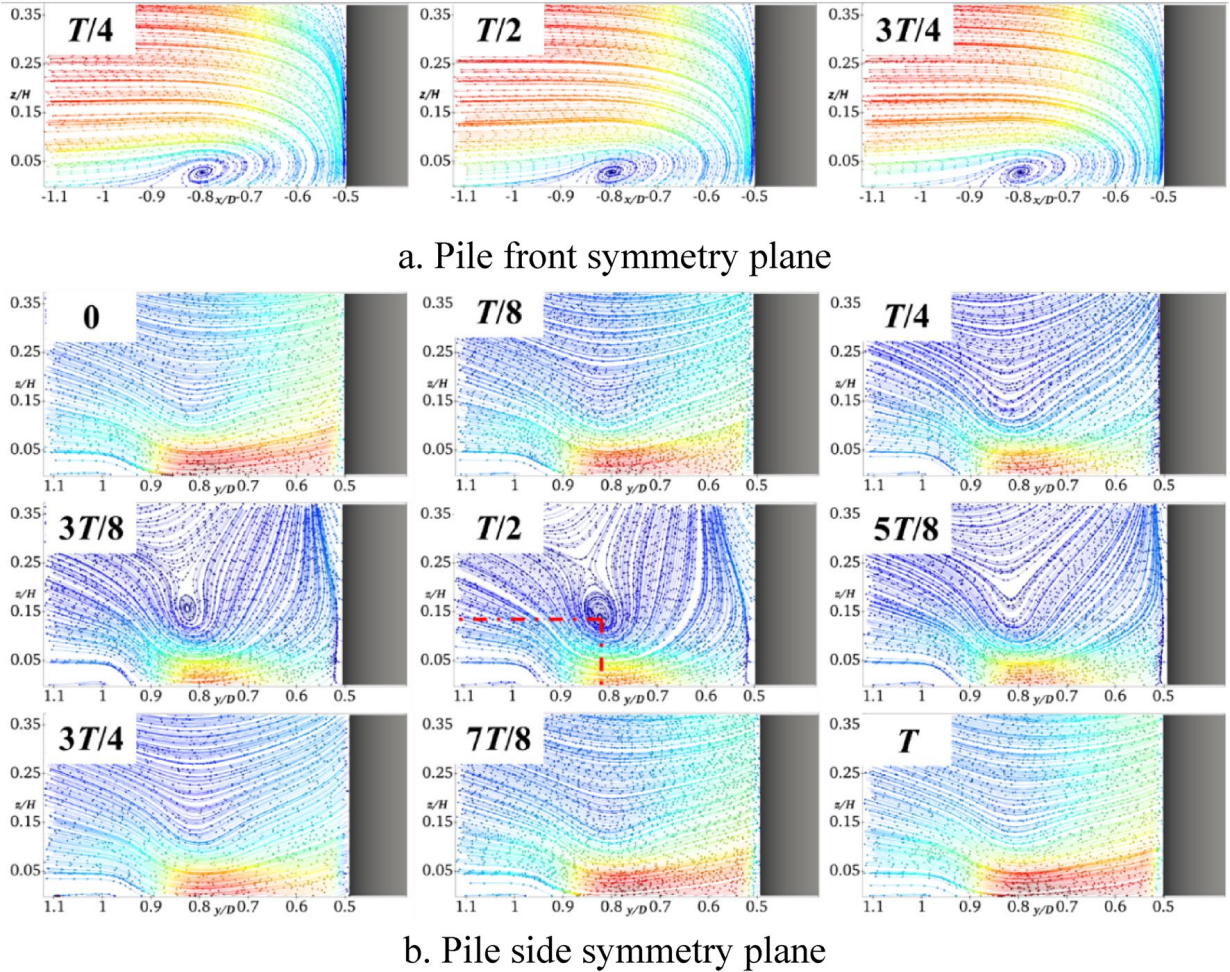
Streamline diagram of YA5f2 at different times (this figure was generated using ParaView (version 5.11.1, URL: https://www.paraview.org)).

Figure 6 shows the results obtained when the pile foundation undergoes lateral oscillation with *A* = 5 mm and *f* = 2 Hz. By comparing Figs. 6 and 10, it can be deduced that the horseshoe vortex and water flow within the symmetry plane in front of the pile are basically similar to those of the stationary pile foundation. In the symmetry plane of the pile side, the water flow is hindered and squeezed by the pile foundation at the moment of *T*/4, and the water flow velocity becomes smaller. When the pile foundation oscillates in the negative *y*-axis direction (*T*/4-*T*/2), there is a large water surface difference and a large amount of descending flow near it. Affected by the shear layers and the descending flow, a clear horseshoe vortex topology occurs in the symmetry plane of the pile side, the center of the horseshoe vortex is located in the pile side with *y*/*D* = 0.82 and *z*/*H* = 0.12, and its topology reaches the maximum at the moment of *T*/2. At the moment of 3*T*/4, the water flow in the symmetry plane of the pile side becomes smaller, and there is no obvious horseshoe vortex topology. In the whole oscillation cycle, when the horseshoe vortex topology periodically changes, the flow velocity near the bed surface also periodically changes, and the flow velocity is minimized at the moment of *T*/2.

**Fig. 11:**
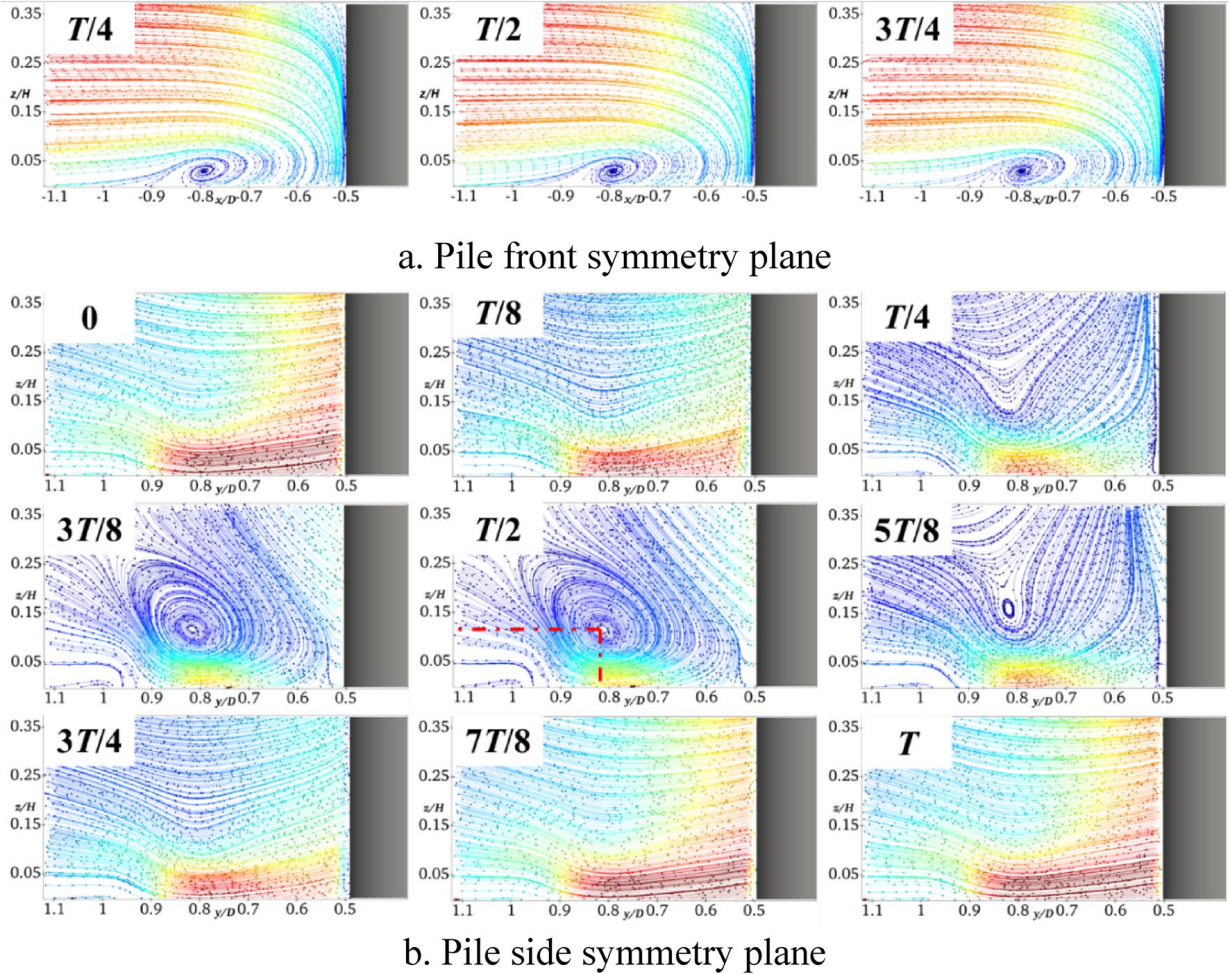
Streamline diagram of YA5f4 at different times.

Figure 11 shows the results obtained for *A* set to 5 mm and *f* increased to 4 Hz. It can be seen that the frequency increase basically does not affect the horseshoe vortex and water flow in the symmetry plane in front of the pile. The horseshoe vortex topology in the symmetry plane on the pile side at the moment of *T*/2 significantly increases, which results in reducing the flow velocity close to the bed. In addition, the center of the horseshoe vortex is still located at approximately *y*/*D* = 0.82 and *z*/*H* = 0.12 on the pile side, which shows that the location of its topology is not sensitive to the frequency change.

**Fig. 12:**
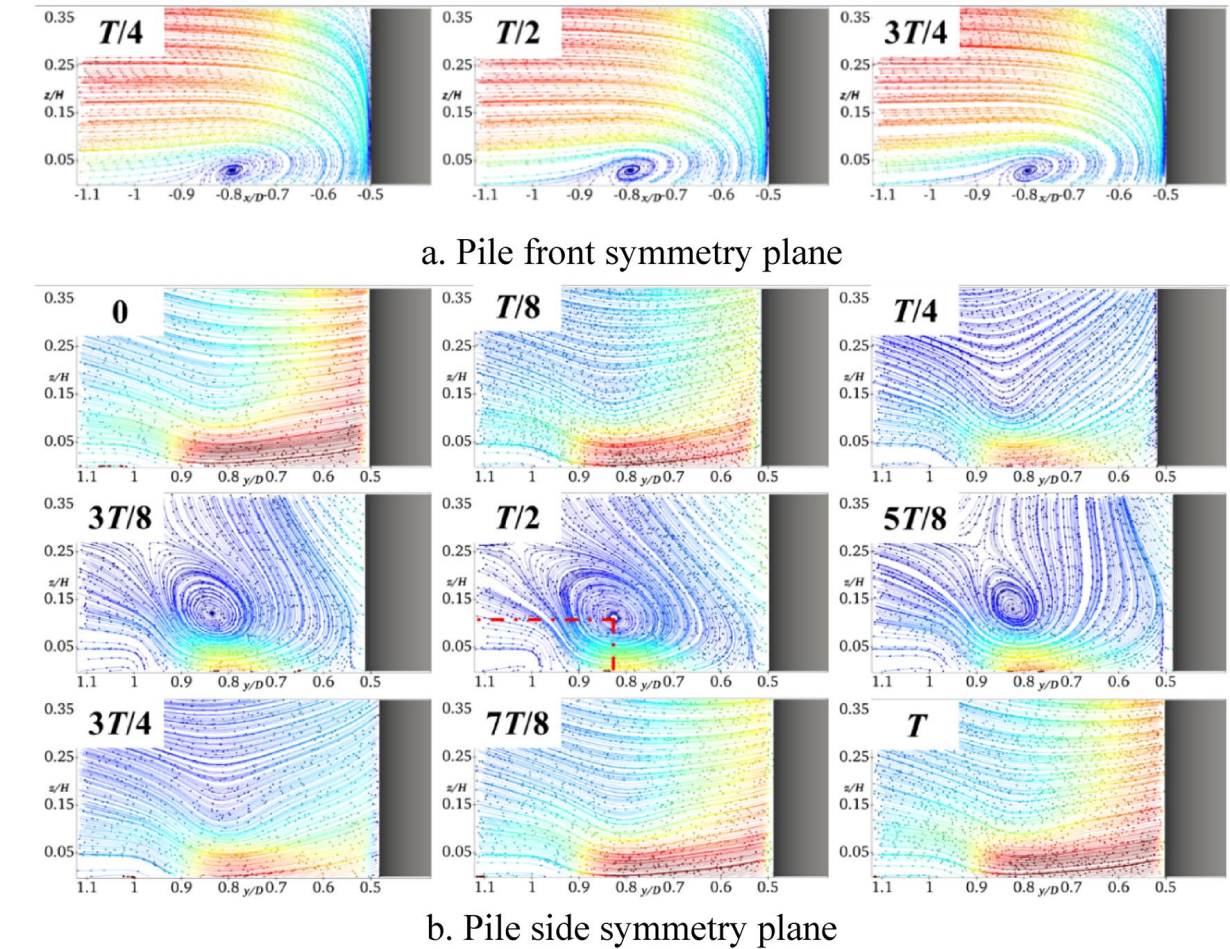
Streamline diagram of YA10f2 at different times.

Figure 12 shows the results obtained for *f* = 2 Hz and *A* = 10 mm. In contrast to the results shown in Fig. 10, the horseshoe vortex and water flow in the symmetry plane in front of the pile are also not affected by the oscillation amplitude. For the symmetry plane on the pile side, the amplitude increase intensifies the obstruction and extrusion of the water flow, and the horseshoe vortex topology significantly increases at the moment of *T*/2. The helix center of the horseshoe vortex basically remains unchanged, which indicates that the location of its topology is insensitive to the amplitude. Similarly, the larger the topology, the smaller the flow velocity close to the bed surface.

### Bed shear stress analysis of transverse and longitudinal oscillatory pile

#### Bed shear stress analysis of longitudinally oscillating pile foundations

**Fig. 13:**
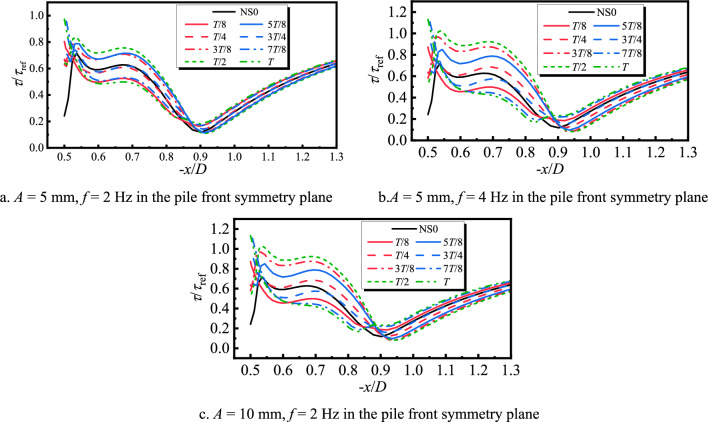
Bed shear stress amplification coefficient curve of longitudinal oscillating pile foundation during period.

The bed shear stress is a key factor for determining the local scour of pile foundation. The horseshoe vortex will enhance the local bed shear stress, and the extent of its change is an important reference index for determining the impact of the lateral and longitudinal oscillation of the pile foundation on local scour^8^. Figure 13 shows the bed shear stress curves on the symmetric plane in front of the pile at different moments in one cycle of the longitudinal oscillation pile foundation, where the vertical coordinate is the dimensionless shear stress amplification factor, and *τ*_ref_ is the bed shear stress value at − *x*/*D* = 2.5.

It can be clearly seen from Figs. 7a, 8a, and 9a that in front of the pile − *x*/*D* = 0.55, the longitudinal oscillation of the pile base produces a large amount of falling water flow and backflow, the velocity gradient greatly varies compared with the stationary one, and the shear stress on the bed surface increases. Horseshoe vortex exists from − *x*/*D* = 0.55 to 0.9, which is the main range of local scouring, and there is a separating saddle point at − *x*/*D* = 0.9 when the shear stress reaches its minimum value. After − *x*/*D* = 0.9, the water flow becomes gradually close to the free-coming flow, and the shear stress amplification factor gradually increases and reaches 1. At the moment of *T*/2, the horseshoe vortex topology is at the maximum, and the flow velocity close to the bed is the largest. Therefore, the shear stress amplification factor corresponding to the moment of *T*/2 moment in Fig. 13 is the largest one. Centered on the moment of *T*/2, the shear stress sequentially decreases in the moments before and after. Increasing the frequency or amplitude will increase the shear stress. The amplification factor of the shear stress at the moment of *T*/2 with *A* = 5 mm and *f* = 2 Hz increases by almost 20% compared with that of the stationary pile foundation. When *f* increases to 4 Hz, the amplification factor of the shear stress at the moment of *T*/2 increases by approximately 50%. For a constant value of *f* of 2 Hz, when *A* increases to 10 mm, the shear stress amplification factor of the moment of *T*/2 increases by approximately 60%.

#### Bed shear stress analysis of transversely oscillating pile foundations

**Fig. 14:**
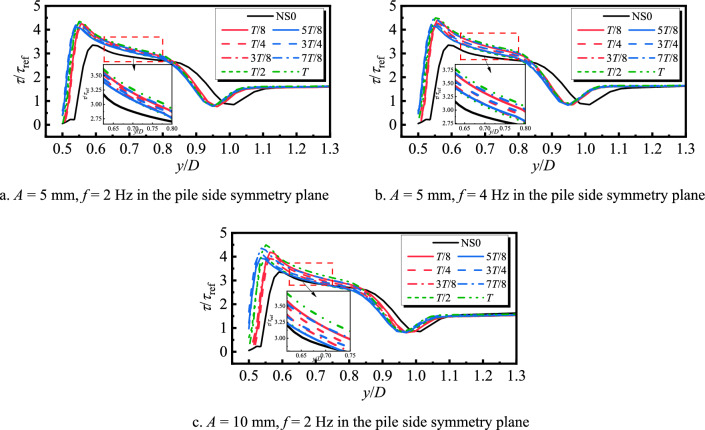
Bed shear stress amplification coefficient curve of transversely oscillating pile foundation during period.

Figure 14 shows the bed shear stress amplification coefficient curves of the symmetry plane of the pile side at different moments during one cycle of the transversely oscillating pile foundation. It can be seen that within the *y*/*D* range of 0.5–0.85 on the pile side, the shear stress is greater than the stationary pile base throughout the transverse oscillation cycle. In addition, when the frequency and amplitude increase, the shear stress also increases, and therefore the transverse oscillation will intensify the local scour on the pile side. It can be observed from Figs. 10b, 11b, and 12b that the lateral oscillation of the pile base intensifies the extension of the horseshoe vortex system on the pile side. Moreover, there is an obvious horseshoe vortex topology in the symmetry plane on the pile side, which reaches the maximum at the moment of *T*/2. However, at this time, the flow velocity close to the bed and the shear stress on the bed are the smallest. The shear stress sequentially increases before and after the moment of *T*/2, and the bed shear stress reaches the maximum at the moment of *T*.

#### Time-averaged bed shear stress analysis of transversely and longitudinally oscillating pile foundations

**Fig. 15:**
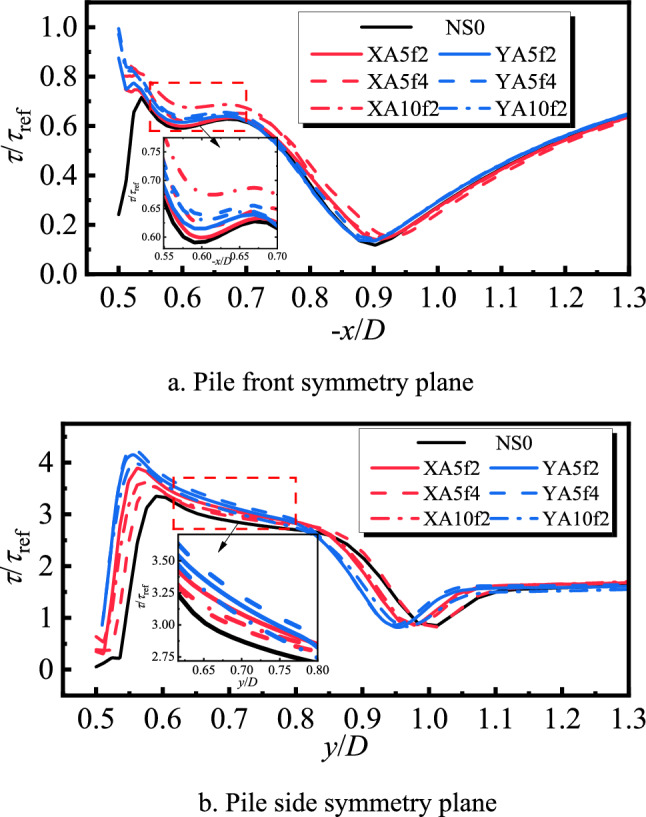
Average bed surface shear stress amplification factor curve for each working condition.

To study the impact of the transversely and longitudinally oscillating pile foundation on the local scour, time-averaged calculations were performed for each moment in the cycle. The obtained graph of time-averaged shear stress amplification factor is shown in Fig. 15. It can be seen from Fig. 15a that the bed shear stresses of the transversely and longitudinally oscillating pile foundations in the symmetry plane in front of the pile at − *x*/*D* between 0.5 and 0.9 are greater than those of the stationary pile foundations. For the longitudinal oscillation, the horseshoe vortex in front of the pile is sufficiently replenished, its topology increases, and there is an obvious reflux. Therefore, the time-averaged shear stress at *A* = 5 mm and *f* = 2 Hz increases by 0.75% compared with that at rest. When *A* remains equal to 5 mm and *f* increases to 4 Hz, the reflux region significantly increases, and the flow velocity increases near the bed surface. This results in increasing the shear stress by 3%. When *f* and *A* respectively remain equal to 2 Hz and 10 mm, the horseshoe vortex topology and the flow velocity near the bed surface increase. Thus, the shear stress increases by almost 10%. It can be seen from the flow line diagrams in Figs. 10a, 11a, and 12a that the transverse oscillation basically does not change the flow on the symmetry surface in front of the pile. However, it produces a water surface difference on the pile side, which leads to the intensification of the flow velocity change in front of the pile and the increase of the velocity gradient. This results in increasing the shear stress in front of the pile. In particular, the shear stress at *A* = 5 mm and *f* = 2 Hz increases by almost 2.5% compared with the stationary time. The shear stress at *A* = 5 mm and *f* = 4 Hz increases by almost 5% and that at *A* = 10 mm and *f* = 2 Hz increases by almost 4%.

It can be observed from Fig. 15b that the bed shear stresses of the transversely and longitudinally oscillating pile foundation within the symmetry plane of the pile side *y*/*D* in the range of 0.5–0.85 are greater than those of the stationary pile foundation. It can be seen from Figs. 7b, 8b, and 9b that the longitudinal oscillation basically does not change the symmetric plane of the water flow on the pile side. However, the resulting accelerated water flow on the pile side results in increasing the velocity gradient, which increases the bed shear stress. However, due to the water viscosity effect and hysteresis phenomenon, for *A* = 5 mm, the shear stress only increases by almost 3.2% and 4.7% for *f* = 4 Hz and 2 Hz, respectively. Compared with the stationary pile foundation, the symmetry surface of the pile side under lateral oscillation shows obvious horseshoe vortex topology, which increases with the increase of the frequency. This results in increasing the velocity gradient near the bed surface and bed shear stress. More precisely, compared with the stationary pile base, the shear stress increases by almost 10% for *A* = 5 mm and *f* = 2 Hz, 12% for a constant *A* of 5 mm and *f* increased to 4 Hz, and 6.5% for a constant *f* of 2 Hz and *A* increased to 10 mm.

## Discussion

Bed shear stress is the tangential force exerted by a fluid on the surface of a riverbed or seabed, whose magnitude directly determines whether sediment particles will be entrained by the flow. It is thus regarded as the core driving factor for local scour around pile foundations. When water flows past a pile foundation, the surrounding flow field undergoes significant distortion, forming complex vortex systems such as horseshoe vortices and wake vortices. These vortices substantially increase the bed shear stress around the pile, particularly at the front and sides, where localized shear stress can reach several times that of the incoming flow^15^. Once the shear stress exceeds the critical incipient stress of sediment (defined by the Shields criterion), particles begin to detach from the bed, leading to the initiation and expansion of scour holes. Both experiments and numerical simulations demonstrate that the depth and extent of scour holes closely correlate with the spatial distribution of bed shear stress around the pile. The scouring process continues until the bed shear stress around the pile decreases below the critical threshold^20^.

This study reveals that transverse and longitudinal oscillations of pile foundations under varying amplitudes and frequencies amplify bed shear stress at the front and sides of the pile. This indicates that oscillating piles are more prone to local scour compared to stationary ones, as their surrounding shear stresses more readily exceed the sediment’s critical incipient stress. Additionally, the bed shear stress on the sides of the pile is found to exceed that at the front, suggesting that scour initiation preferentially occurs along the pile sides.

Local scour around oscillating piles is governed by the coupling between oscillation intensity and hydrodynamic conditions. Yao et al.^25^ classified scour patterns around oscillating piles under different hydrodynamic conditions into three regimes:

- Regime 1: Under relatively weak hydrodynamic intensity, waves and currents exhibit limited influence on scour hole development around both static and vibrating monopiles. The settlement motion of sand around vibrating piles drives further scour progression. In this regime, scour holes around vibrating monopiles are larger than those around static foundations, as observed in Yao et al.'s^30^ research.
- Regime 2: Both vibration and hydrodynamic factors exert comparable and non-negligible influences on local scour. Different combinations of vibration parameters and hydrodynamic conditions may yield varying scour hole dimensions. The characteristic dimensions of equilibrium scour holes around vibrating monopiles fluctuate within ± 10.9% of those under static conditions.
- Regime 3: Strong hydrodynamic intensity (as in Scenario 3) induces larger scour holes around monopiles. Here, sand settlement depth becomes negligible compared to scour depth. However, convective sand motion around vibrating piles promotes particle migration toward scour hole bottoms, hindering sediment transport out of the pit. Consequently, shallower and smaller scour holes form around vibrating monopiles, as reported in studies by Al-Hammadi and Simons^23^ and Guan et al.^22^.

Compared to static foundations, varying combinations of vibration parameters and hydrodynamic conditions may lead to divergent scour hole dimensions. The deviations in scour dimensions around vibrating monopiles underscore the need to modify traditional equilibrium scour depth prediction formulas to account for vibration-induced scour mechanisms. While this study does not explicitly investigate scour depth prediction, it elucidates hydrodynamic characteristics, bed shear stress variation mechanisms, and scour-prone zones around oscillating piles, laying groundwork for improved predictive formulas.

It should be noted that this research focuses on clear-water scour conditions. Future studies could develop air–water-sediment three-phase coupled live-bed numerical simulations to investigate bed shear stress under evolving scour hole dimensions, enabling more accurate predictions of scour depth around oscillating piles.

## Conclusion

This paper studies the vortex structure and bed shear stresses in the symmetry plane in front of the pile and on its side at different frequencies and amplitudes of its foundation. This is performed to evaluate the impact of the oscillating pile foundation on its local scouring. The main conclusions can be summarized as follows:

1. Transversely and longitudinally oscillation increases bed shear stresses around the pile, and is remarkably affected by the frequency and amplitude of the oscillations.
2. In the oscillation cycle, the longitudinal oscillation of the pile foundation in front of the pile symmetry plane produces an obvious reflux, and more backflow is generated by increasing the frequency and amplitude of oscillation. At the moment of *T*/2, the horseshoe vortex reaches its maximum value and the bed shear stress is the largest, which does not affect the symmetry plane of the pile side of the water flow.
3. In the oscillation cycle, lateral oscillation pile base will extend the pile side horseshoe vortex system, and an increase in the oscillation frequency and amplitude will make the horseshoe vortex increased at the pile side. At the moment of *T*/2, the horseshoe vortex reaches its maximum value and the bed shear stress reaches its minimum value. At the moment of *T*, the bed shear stress reaches its maximum value. This does not affect the symmetry plane in front of the pile water flow.
4. Both transverse and longitudinal oscillations increase the time-averaged bed shear stresses. The values created by longitudinal oscillations are larger than those created by transverse oscillations in the front of the pile. This tendency is changed at the pile side, where the transverse oscillations generated larger time-averaged bed shear stresses.

The limitations of this study and proposals for future research are as follows:

1. The sea bed is set as a fixed flat condition in this study without considering the shape change due to soil moving. While, numerical studies can be carried out based on shape-unfixed beds considering air–water-soil three-phase flow in the future.
2. This study focuses on the fluid dynamics around a vibrating pile, and does not investigate scour development under the dynamic flow influence. A more in-depth study can be conducted in the future by further considering the induced motion of sand (settlement or convection).

## Data Availability

All data generated or analyzed during this study are included in this published article.

## List of symbols

CFD: Computational fluid dynamics
VOF: Volume of fluid
SST: Shear stress transport
*x*: *x*-direction
*y*: *y*-direction
*z*: *z*-direction
*T*: Time
*α*: Volume fraction
*μ*: Kinetic viscosity coefficient of liquid mixture
*ρ*: Density of the liquid mixture
*D*: Diameter of the pile foundation
*U*_m_: Velocity of the incoming current
*H*: Water depth
*A*: Amplitude
*F*: Frequency
*τ*: Bed shear stress ratio
*τ*_ref_: Reference value of bed shear stress ratio
